# Immunosuppression by Mycophenolate Mofetil Mitigates Intrarenal Angiotensinogen Augmentation in Angiotensin II-Dependent Hypertension

**DOI:** 10.3390/ijms23147680

**Published:** 2022-07-12

**Authors:** Ryousuke Satou, Martha Franco, Courtney M. Dugas, Akemi Katsurada, L. Gabriel Navar

**Affiliations:** 1 Department of Physiology and Hypertension and Renal Center of Excellence, Tulane University School of Medicine, New Orleans, LA 70112, USA; cdugas1@tulane.edu (C.M.D.); asato@tulane.edu (A.K.); navar@tulane.edu (L.G.N.); 2 Departments of Nephrology and Pathology, Instituto Nacional de Cardiologia, Mexico City 14080, Mexico; marthafranco@lycos.com

**Keywords:** angiotensinogen, angiotensin II, mycophenolate mofetil, kidney injury, hypertension

## Abstract

Augmentation of intrarenal angiotensinogen (AGT) leads to further formation of intrarenal angiotensin II (Ang II) and the development of hypertensive kidney injury. Recent studies demonstrated that macrophages and the enhanced production of pro-inflammatory cytokines can be crucial mediators of renal AGT augmentation in hypertension. Accordingly, this study investigated the effects of immunosuppression by mycophenolate mofetil (MMF) on intrarenal AGT augmentation. Ang II (80 ng/min) was infused with or without daily administration of MMF (50 mg/kg) to Sprague-Dawley rats for 2 weeks. Mean arterial pressure (MAP) in Ang II infused rats was slightly higher (169.7 ± 6.1 mmHg) than the Ang II + MMF group (154.7 ± 2.0 mmHg), but was not statistically different from the Ang II + MMF group. MMF treatment suppressed Ang II-induced renal macrophages and IL-6 elevation. Augmentation of urinary AGT by Ang II infusion was attenuated by MMF treatment (control: 89.3 ± 25.2, Ang II: 1194 ± 305.1, and Ang II + MMF: 389 ± 192.0 ng/day). The augmentation of urinary AGT by Ang II infusion was observed before the onset of proteinuria. Elevated intrarenal AGT mRNA and protein levels in Ang II infused rats were also normalized by the MMF treatment (AGT mRNA, Ang II: 2.5 ± 0.2 and Ang II + MMF: 1.5 ± 0.1, ratio to control). Ang II-induced proteinuria, mesangial expansion and renal tubulointerstitial fibrosis were attenuated by MMF. Furthermore, MMF treatment attenuated the augmentation of intrarenal NLRP3 mRNA, a component of inflammasome. These results indicate that stimulated cytokine production in macrophages contributes to intrarenal AGT augmentation in Ang II-dependent hypertension, which leads to the development of kidney injury.

## 1. Introduction

Hypertension accounts for approximately one fourth of all heart failure cases, of which 60% are attributed to hypertension in the elderly [1]. The most common causes of end-stage renal disease in the United States are hypertension and diabetes. Systemic and local renin-angiotensin systems (RAS) play crucial roles in controlling blood pressure and regulating electrolyte and body fluid homeostasis [2]. Since inappropriate activation of the intrarenal RAS, which increases intrarenal angiotensin II (Ang II) formation, appears to play a crucial role in the progression of hypertension and associated kidney injury [2,3], mechanisms underlying regulation of intrarenal RAS components have been investigated. In the kidneys of Ang II-infused animals and human renin/human AGT double-transgenic mice, elevated intrarenal angiotensinogen (AGT) expression and urinary AGT levels were observed [4,5,6], supporting an intrarenal Ang II-AGT amplifying mechanism in Ang II-dependent hypertension. Thus, augmentation of renal proximal tubular AGT has been regarded as one of the key mechanisms leading to enhanced intrarenal Ang II production in Ang II-dependent hypertension [2,3]. However, some in vitro studies using cultured human renal proximal tubular cells (PTC) demonstrated that maximum AGT upregulation by treatment with Ang II requires the presence of cytokines or co-factors such as interleukin 6 (IL-6) [7,8]. These findings suggest that a mediator and/or co-factors are required for intrarenal AGT augmentation observed in Ang II-dependent hypertension.

Studies have demonstrated intimate links between inflammation and the regulation of hypertension in both human and experimental animal models [9,10]. Among the wide-ranging actions of the RAS, its role in immune-promoting hypertension development has come into focus [11,12,13]. Ang II induces differentiation of immune cells and augmentation of pro-inflammatory cytokine production [14,15,16,17], both contributing to elevated blood pressure and sustained hypertensive conditions. The increases in intrarenal immune cells and pro-inflammatory cytokines have been observed in Ang II-dependent hypertension [18,19]. Furthermore, the expression of NLRP3, which is a component of inflammasome, is augmented by Ang II in renal cells and in kidneys of chronic Ang II-induced hypertension models [20,21,22,23,24,25]. In vitro studies using cultured PTC have provided evidence that elevated pro-inflammatory cytokines contribute to AGT upregulation [7,8,26,27]. In particularly, a study using a sequencing cell-culture system showed that macrophages activated by Ang II treatment and, consequently, over-produced IL-6 augment AGT expression in PTC [27]. Accordingly, an activated immune system can serve as an important mediator of augmentation of proximal tubular AGT in Ang II-dependent hypertension. However, the roles of the immune system in intrarenal AGT regulation under in vivo hypertensive conditions have not been established. Administration of mycophenolate mofetil (MMF), an immunosuppressive drug, reduced intrarenal Ang II in a lead-induced hypertensive animal model [28]. Therefore, we performed this study to test our hypothesis that immunosuppression by MMF attenuates intrarenal AGT augmentation and development of hypertensive kidney injury in Ang II-induced hypertension rats.

## 2. Results

### 2.1. Effects of MMF on Blood Pressure

**Figure 1:**
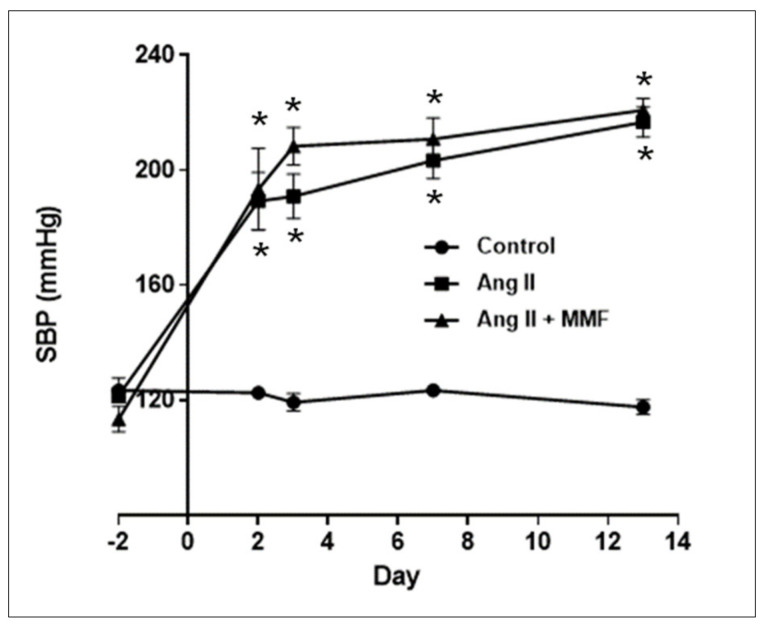
Effects of MMF on blood pressure. SBP was measured by a tail-cuff plethysmography system on day −2, 2, 3, 7 and 13. Mean ± SE. *n* = 6 in each group. Asterisk (*p* < 0.05) indicates significant difference compared to the control group at each time point.

Chronic Ang II infusion increased SBP from 121.7 ± 2.5 to 189.2 ± 10.0 mmHg by day 2 and 216.7 ± 5.3 mmHg by day 13 (Figure 1, *n* = 6 in each group). Ang II-infused rats treated with MMF exhibited similar increases in SBP (220.8 ± 4.2 mmHg on day 13).

### 2.2. Effects of MMF on GFR and Mean Arterial Pressure (MAP)

Glomerular function was evaluated using inulin clearance. Rats subjected to chronic Ang II infusion had slightly lower GFR (0.90 ± 0.03, *n* = 6) compared to the control group (1.07 ± 0.04, *n* = 6). MMF treatment of Ang II-infused rats did not significantly alter GFR values (1.11 ± 0.04 in the Ang II + MMF group). MAP measured during the clearance period showed significantly increased values in Ang II-infused group (126.7 ± 1.3 mmHg in the control group vs. 169.7 ± 6.1 mmHg in Ang II-infused group). The MMF-treated group had slightly lower MAP (156.9 ± 2.0 mmHg).

### 2.3. Effects of MMF on Intrarenal Immune Cells and IL-6 Levels

**Figure 2:**
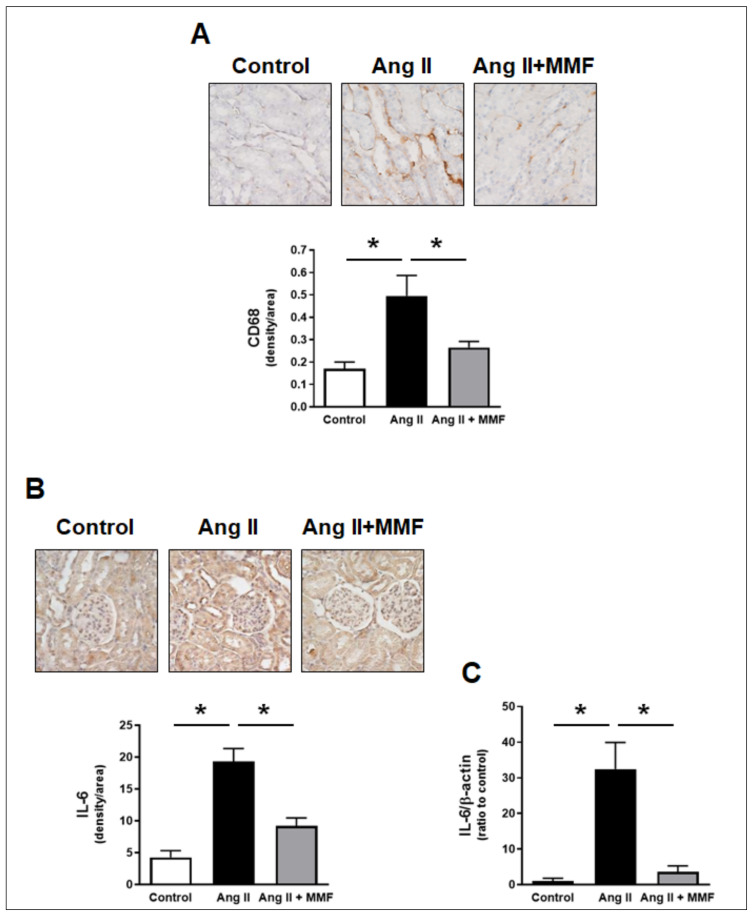
Effects of MMF on intrarenal immune cells and IL-6 levels. Quantified scores of positive areas for CD68 (monocyte/macrophage staining) and IL-6 protein and representative images are shown in panels (**A**,**B**), respectively, (*n* = 6. The brown color indicates positive area. The bar graphs show the percentile of density of the positive area in the total image). The scoring method for the images are described in the Method section. Panel (**C**) indicates renal cortical IL-6 mRNA levels (*n* = 6). Asterisk (*p* < 0.05) indicates significant difference between groups.

Levels of intrarenal monocyte/macrophage were measured by CD68 staining. Chronic Ang II infusion increased the density of CD68 positive area in tubulointerstitium of the renal cortex (Figure 2A, 0.169 ± 0.03 in the control group vs. 0.495 ± 0.09 in Ang II-infused group, arbitrary density units, *n* = 6). In addition, the density of the IL-6 positive area in the renal cortex was concomitantly augmented by the chronic Ang II infusion (Figure 2B, 4.27 ± 1.03 in the control group vs. 19.3 ± 2.01 in Ang II-infused group, arbitrary density unit, *n* = 6). Intrarenal IL-6 mRNA levels were also markedly greater in the Ang II-infused group than in the control group (Figure 2C, 32.4 ± 7.44-fold in the Ang II-infused group, ratio to the control group, *n* = 6). The greater values of intrarenal monocyte/macrophage, IL-6 mRNA and protein in the Ang II-infused group were prevented by MMF treatment (Figure 2A–C).

### 2.4. Effects of MMF on Intrarenal and Urinary AGT Levels

**Figure 3:**
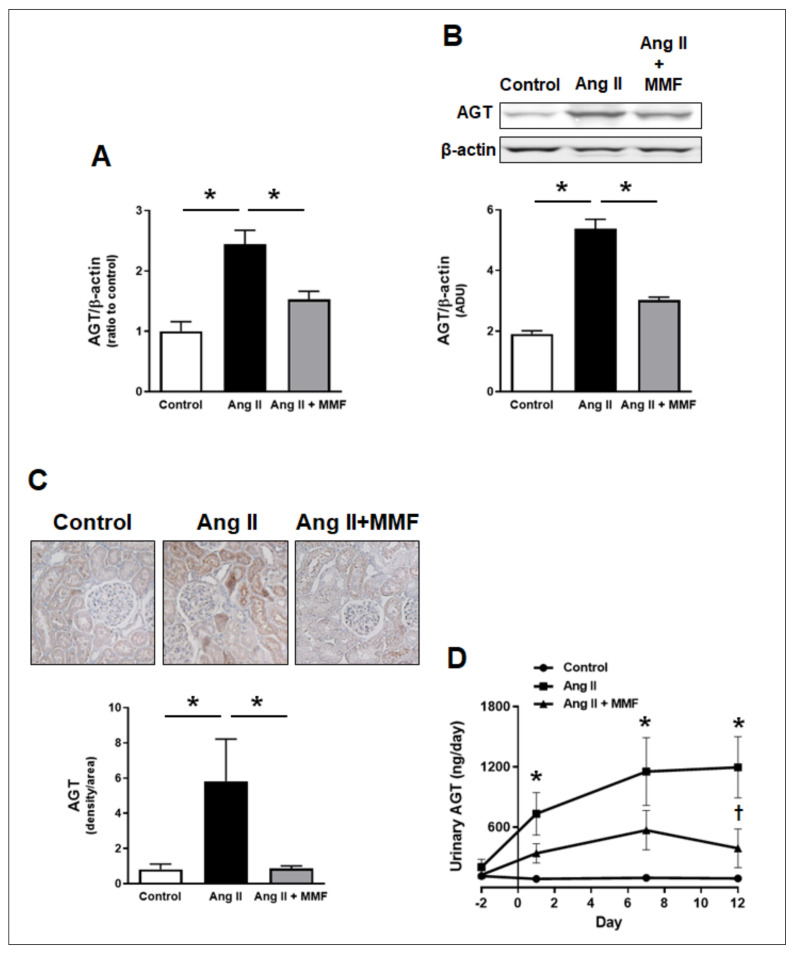
Effects of MMF on intrarenal and urinary AGT levels. Panel (**A**) indicates renal cortical AGT mRNA levels (*n* = 6). Panels (**B**,**C**) show renal cortical AGT protein levels detected by Western blot analyses and immunological staining, respectively (*n* = 6). The brown color in the images indicates positive areas. The bar graphs show the percentile of density of positive areas in the total image. The scoring method for the images is described in the Method section. Urinary AGT levels determined by an ELISA are presented in panel (**D**) (*n* = 6). In panels (**A**–**C**), an asterisk (*p* < 0.05) indicates significant difference between the groups. In panel (**D**), an asterisk (*p* < 0.05) indicates significant difference compared to the control group and the dagger (*p* < 0.05) shows significant difference between Ang II-infused and Ang II + MMF groups.

As mentioned, renal proximal tubular AGT regulation has been shown to play crucial roles in the development of kidney injury in hypertension. Thus, renal cortical AGT mRNA and protein levels were evaluated. Renal cortical AGT mRNA levels in Ang II-infused group were 2.44 ± 0.23-fold greater than in the control group (Figure 3A, *n* = 6). Western blot analysis showed that renal cortical AGT protein levels were also increased in Ang II-infused group (Figure 3B, 1.91 ± 0.10 in the control group vs. 5.83 ± 0.32 in Ang II-infused group, arbitrary density unit, *n* = 6). In immunological staining of AGT, AGT protein was detected mainly in proximal tubules as previously demonstrated in rat kidneys [29,30]. Results obtained by immunological staining support the finding in Western blot analysis indicating augmentation of renal cortical AGT protein in Ang II-dependent hypertension (Figure 3B, 0.81 ± 0.31 in the control group vs. 5.82 ± 2.40 in Ang II-infused group, arbitrary density units, *n* = 6). Importantly, MMF treatment attenuated the increases in AGT mRNA and protein levels in the renal cortex of Ang II-infused rats (Figure 3A–C).

It has been proposed that urinary AGT levels reflect intrarenal AGT production levels and serve as a marker of hypertensive kidney injury during the early stage of the injury [31,32,33]. There are no differences in urinary AGT levels among the three groups at baseline (Figure 3D, *n* = 6). Urinary AGT levels were increased, even on day 1 of Ang II infusion (84.5 ± 16.4 in the control group vs. 732.5 ± 212.2 in Ang II-infused group on day 1, AGT ng/day, *n* = 6). In Ang II-infused group, urinary AGT levels were increased further on day 7 (1153.1 ± 338.2 in Ang II-infused group on day 7, AGT ng/day, *n* = 6) and the elevated levels were sustained until day 12 (1194.6 ± 305.1 in Ang II-infused group on day 12, AGT ng/day, *n* = 6). A group receiving Ang II infusion and MMF treatment exhibited a lesser elevation of urinary AGT levels (Ang II + MMF group: 339.1 ± 96.9 on day 1, 569.8 ± 197.0 on day 7 and 389.6 ± 192.0 on day 12, AGT ng/day, *n* = 6); however, these values are not statistically different compared to the control group at each time point (one-way ANOVA followed by post hoc Tukey multiple comparison test). The urinary AGT levels in Ang II + MMF-treated group was significantly lower than in Ang II-infused group on day 12.

### 2.5. Effects of MMF on Hypertensive Kidney Injury

**Figure 4:**
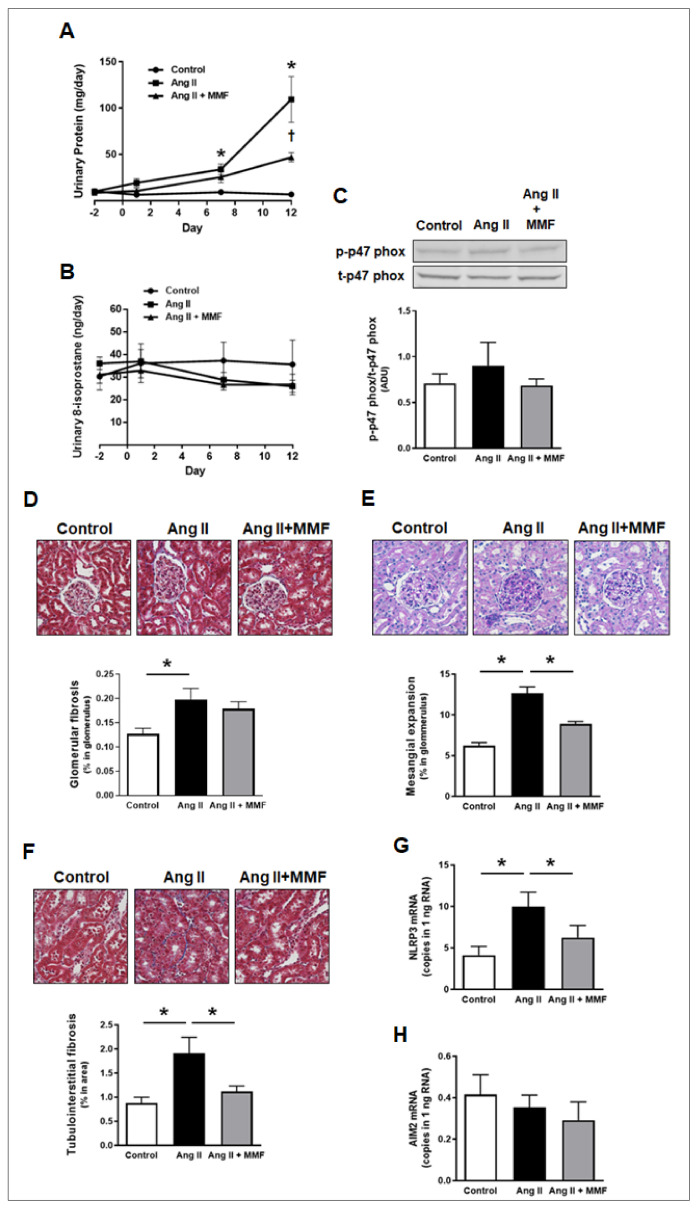
E Effects of MMF on hypertensive kidney injury. Panel (**A**) indicates urinary protein levels (*n* = 6). Panels (**B**,**C**) show markers of renal oxidative stress, urinary 8-isoprostane and renal cortical phosphor-p47 phox levels, (*n* = 6). Panels (**D**–**F**) are scores and representative pictures of glomerular fibrosis (MT staining, blue color indicates positive area. The bar graph shows the percentile of blue positive area in the total image or glomerulus.), glomerular mesangial expansion (PAS staining, pink color indicates positive area. The bar graph shows the percentile of pink positive area in the glomerulus.) and tubulointerstitial fibrosis (MT staining), respectively (*n* = 6). Panels (**G**,**H**) indicate mRNA copy numbers of NLRP3 and AIM2 in the renal cortex detected by ddPCR (*n* = 6). Asterisk (*p* < 0.05) indicates significant difference between groups.

Ang II-infused rats developed proteinuria. Urinary protein levels in the group were significantly greater than in the control group by day 7 (Figure 4A, 9.0 ± 1.4 in the control group vs. 33.7 ± 5.7 in Ang II-infused group on day 7, mg/day, *n* = 6) and day 12 (6.7 ± 1.6 in the control group vs. 109.3 ± 24.6 in Ang II-infused group on day 12, mg/day, *n* = 6). MMF treatment prevented the development of proteinuria by Ang II infusion compared to the control group and the levels were significantly lower than in Ang II-infused group on day 12 (Ang II + MMF group: 46.7 ± 5.1 on day 12, mg/day, *n* = 6).

Urinary 8-isoprostane levels were measured to evaluate the development of renal oxidative stress. The urinary 8-isoprostane levels were not statistically different among the three groups during the tested period (Figure 4B, *n* = 6). To evaluate further, we measured phosphorylation levels of renal cortical p47 phox, an indicator of tissue oxidative stress, by Western blot analysis. The phosphorylation levels were not altered by either Ang II infusion or MMF treatment during the 14 day period (Figure 4C, *n* = 6).

Glomerular and cortical tubular damage was evaluated by histological analyses. Glomerular fibrosis, which was detected by MT staining, was induced by Ang II infusion (Figure 4D, 0.127 ± 0.01 in the control group vs. 0.198 ± 0.02 in Ang II-infused group, % positive area in glomerulus, *n* = 6). MMF treatment did not prevent the development of glomerular fibrosis (0.178 ± 0.02 in the Ang II + MMF group, % positive area in glomerulus, *n* = 6). Conversely, MMF treatment attenuated glomerular mesangial expansion, indicated by PAS staining, in Ang II-infused rats (Figure 4E, 6.21 ± 0.38 in the control group, 12.66 ± 0.78 in Ang II-infused group and 8.90 ± 0.29 in the Ang II + MMF group, % positive area in glomerulus, *n* = 6). Ang II infusion elicited tubulointerstitial fibrosis (Figure 4F, 0.88 ± 0.12 in the control group vs. 1.91 ± 0.33 in Ang II-infused group, % positive area in glomerulus, *n* = 6), which was attenuated by MMF treatment (1.12 ± 0.11 in the Ang II + MMF group, % positive area in glomerulus, *n* = 6).

The levels of NLRP3 and AIM2 mRNA, inflammasome-associated genes, were determined to investigate the development of renal inflammation. As mentioned, ddPCR technique was employed due to relatively low amounts of these target genes. NLRP3 mRNA levels in the renal cortex of Ang II-infused rats were significantly higher than in the cortex of the control group (Figure 4G, 4.12 ± 0.96 in the control group vs. 9.96 ± 1.62 in the Ang II-infused group, mRNA copies in 1 ng RNA, *n* = 6). Rats receiving Ang II infusion in the presence of MMF treatment did not show significant augmentation of NLRP3 mRNA expression (6.24 ± 1.32 in the Ang II + MMF group, mRNA copies in 1 ng RNA, *n* = 6). AIM2 mRNA copy numbers in the renal cortex were much lower than NLRP3 levels and not different among the three tested groups (Figure 4H, *n* = 6).

## 3. Discussion

The results obtained in the present study demonstrated that immunosuppression by MMF attenuates intrarenal AGT augmentation in Ang II-dependent hypertension, which is associated with mitigation of proteinuria, mesangial expansion and renal tubulointerstitial fibrosis.

Although a relatively mild dose of Ang II infusion (80 ng/min) was used in this study, the chronic Ang II infusion markedly elevated the SBP of rats, even on day 2, and the SBP was increased further to >200 mmHg after seven days Ang II infusion. Since a previous study demonstrated that MMF treatment suppressed high blood pressure in several hypertensive models and patients, including lead-induced hypertension [28], patients with psoriasis and rheumatoid arthritis [34], DOCA-salt hypertensive rats [35] and spontaneously hypertensive rats [36], attenuation of elevated SBP accompanied by inhibition of intrarenal AGT augmentation by MMF was anticipated in the present study. However, MMF did not exhibit a significant anti-hypertensive effect, which was shown by a tail-cuff plethysmography system. Therefore, it is possible that the ability of MMF to reduce blood pressure depends on the co-existing Ang II levels, but further specific investigations of the anti-hypertensive effects of MMF need to be performed.

Chronic Ang II infusion enhances the number of immune cells in kidneys [18,19], and Ang II has been shown to directly stimulate IL-6 expression in macrophages [27,37]. In the present study, MMF prevented the accumulation of macrophage/monocyte and augmentation of IL-6 mRNA and protein levels in the renal cortex of Ang II-infused rats as expected. Recent studies revealed that there are kidney-resident macrophages derived from embryo, in addition to bone-marrow-derived macrophages in adult kidneys [38,39]. Although the source of macrophages contributing to pro-inflammatory cytokine elevation in the kidneys of Ang II-dependent hypertension has not been identified, production of IL-6, a stimulus of proximal tubular AGT expression [27], has been demonstrated in kidney-resident macrophages [40]. Therefore, both kidney-resident and bone-marrow-derived macrophages may be able to augment renal cortical IL-6 levels in Ang II-dependent hypertension.

The augmentation of renal cortical AGT mRNA and protein in hypertensive rats was prevented by immunosuppression with MMF. Furthermore, elevated urinary AGT levels by Ang II infusion was also suppressed by MMF. The crucial role of elevated IL-6 produced by Ang II-treated macrophages in proximal tubular AGT augmentation was demonstrated in a previous study using sequencing cell culture system [27]. Thus, attenuation of the increases in intrarenal macrophages and IL-6 by MMF may have resulted in the inhibition of renal cortical AGT upregulation and, consequently, elevated urinary AGT levels. Supplemental Figure S1 compares temporal changes in urinary AGT, protein and 8-isoprostane levels during Ang II infusion, which are shown in Figure 3D and Figure 4A,B, respectively. Importantly, significant elevation of urinary AGT levels was observed before the onset of proteinuria (Figure S1). This temporal dissociation indicates that the elevation of intrarenal AGT production, resulting from the urinary AGT excretion rates, occurs in a proteinuria-independent manner. Furthermore, augmentation of urinary AGT can serve as an early marker for hypertensive kidney injury, as previously proposed [2]. Renal oxidative stress has also been identified as a strong enhancer of proximal tubular AGT expression under diabetic conditions [41,42,43,44]. In the present study, however, renal oxidative stress, indicated by urinary 8-isoprostane and phosphorylation of renal cortical p47 phox, was not observed during the 2-week Ang II infusion period. This result supports the previous finding that elevated urinary 8-isoprostane was not observed on day 21 or later in Ang II infused (60 ng/min) rats [45]. Therefore, although renal oxidative stress is a key pathological factor in the late stage of hypertensive kidney injury, the oxidative stress is unlikely to contribute to renal AGT augmentation that occurs during the early stage of Ang II-dependent hypertension. These results suggest that the sequential mechanisms leading to intrarenal AGT augmentation in hypertension and diabetes mellitus are different.

Our histological analyses showed that MMF mitigated the progression of mesangial expansion and renal tubulointerstitial fibrosis in Ang II-infused rats. MMF also attenuated the elevation of renal IL-6 and subsequent renal AGT, suggesting that increased AGT and activated intrarenal RAS participate in the development of these kidney injuries. Importantly MMF did not attenuate the development of glomerular fibrosis in Ang II-dependent hypertension, suggesting that glomerular fibrosis is caused by pathological factors other than elevated IL-6 and augmented renal AGT. Interestingly, MMF suppressed the augmentation of NLRP3 expression in kidneys of Ang II-infused rats, which downregulates NLRP3 inflammasome complex formation. While augmentation of NLRP3 expression by Ang II in renal cells and in kidneys of chronic Ang II-induced hypertension models has been demonstrated [20,21,22,23,24,25], mitigation of the NLRP3 expression by immunosuppression in hypertension is a novel observation. Although NLRP3 inflammasome is activated by ATP-P2Y7 axis and reactive oxygen species, the expression of pro-NLRP3 is promoted by NF-κB activated by cytokines or PAMPs/DAMPs [46,47]. As mentioned previously, the elevation of oxidative stress was not induced during the 2-week Ang II infusion period. Thus, reactive oxygen species might not be a regulator of renal NLRP3 in the present study. MMF suppressed the accumulation of renal macrophages that produce NF-κB activating pro-inflammatory cytokines such as TNF-α and IL-1β. Therefore, MMF may inhibit elevation of NLRP3 via attenuation of these pro-inflammatory cytokines and NF-κB activation in Ang II-dependent hypertension. Future studies will elucidate further these interesting mechanisms. AIM2 forms inflammasome complex by viral or bacterial infections [47]. Thus, no changes in intrarenal AIM2 levels in Ang II-infused rats are expected and the results highlight specific regulation of renal NLRP3 in hypertension. Furthermore, AIM2 mRNA copy numbers in the renal cortex were much lower than NLRP3 mRNA copy numbers in all groups.

In this study, the histological and molecular analyses demonstrated the development of glomerular injury by Ang II infusion. However, differences in inulin clearance values among the groups were not observed. This may be due to partial maintenance of glomerular pressure and GFR by the increases in blood pressure.

In conclusion, this study demonstrated that stimulated IL-6 production in activated macrophages contributes to intrarenal AGT augmentation in the early stages of Ang II-dependent hypertension, which leads to the development of kidney injury. MMF blocks the activation of the sequential pathological cascade.

## 4. Materials and Methods

### 4.1. Animals and Sample Collection

Animal procedures were performed in accordance with the Mexican Federal Regulation for animal experimentation and care (NOM-062-ZOO-2001) and protocols were approved by the Investigation Committee of the Instituto Nacional de Cardiología “Ignacio Chávez”, (INC-CICUAL/012/2019, 17-1142). All rats had free access to water and a standard chow diet.

### 4.2. Induction of Hypertension

Rats were infused with Ang II (Sigma, St Louis, MO, USA) via subcutaneous osmotic minipumps (Alzet model 2002, Alza Corp, Palo Alto, CA, USA) implanted under isoflurane anesthesia. Mini-pumps delivered AngII 80 ng/Kg/min. Experiments were performed 14 days after the implant.

Three groups of rats were studied: Sham operated rats and AngII-infused groups (*n*= 6 per group) received either vehicle or Angiotensin II. The third group consisted of rats (*n* = 6) that received Angiotensin II + mycophenolate mophetil (MMF, 50 mg/kg per day by gastric gavage) during the AngII infusion (AngII-MMF). The MMF was suspended in water by vigorous agitation immediately before administration because it is insoluble in water, as described in previous communications [48]. Systolic blood pressure measurements and 24 h urine collections were taken at −2, 2, 3, 7 and 13 days.

On day 14, the rats were anesthetized with pentobarbital sodium (30 mg.Kg, i.p); the kidneys were harvested and weighed, one kidney was fixed in 4% paraformaldehyde and cortex and medulla were separately collected from the other kidney, frozen in liquid nitrogen and stored at −80 °C until they were processed for molecular biological analyses.

### 4.3. Blood Pressure Measurements

Systolic blood pressure (SBP) measurements were performed in conscious, restrained rats by tail-cuff plethysmography (IITC Life Science). The rats were conditioned before blood pressures were measured at −2, 2, 3, 7 and 13 days.

### 4.4. Urine Assays

Urinary protein and 8-isoprostane levels were determined by Protein Assay Rapid kit (Wako) and an 8-isoprostane EIA kit (Cayman). Urinary AGT levels were measured by AGT ELISA kit (IBL, Minneapolis, MN, USA).

### 4.5. Clearance Experiments

On day 14 after the initiation of the Ang II infusion, the rats were anesthetized with sodium pentobarbital (30 mg/kg, i.p.) and supplementary doses were administered as required. The rats were placed on a thermos-regulated table with the temperature maintained at 37 °C. Polyethylene tubing was used to catheterize the trachea (PE-240), jugular vein, the right femoral artery (PE-50), and the left ureter (PE-10). The left kidney was exposed and placed in a lucite holder, and the kidney surface was bathed with Ringer’s solution. A 6% albumin solution (1% of body weight, Sigma, St Louis, MO, USA) was infused through the jugular catheter. Immediately after, a blood sample was taken and a bolus injection of 100 mg of polyfructosan (Inutest, Fressenius Pharma, Graz, Austria) in 0.5 mL in 0.9% sodium chloride solution was given, following which a sustaining infusion of 10% polyfructosan saline solution was started at a rate of 2.2 mL/h. After a 60 min equilibration period, blood samples were taken before and after a 45–60 min urine sample collection. Blood was replaced with red blood cells resuspended in a sodium chloride solution. Polyfructosan was measured in the plasma and the urine samples, as previously described [49]. Mean arterial pressure (MAP) was continuously monitored via the femoral arterial catheter connected to a pressure transducer (Model p23 LX, Gould. Hato Rey, Puerto Rico) and recorded on a polygraph (Grass Instruments, Quincy, MA, USA).

### 4.6. Real-Time RT-PCR

Quantitative real-time RT-PCR (qRT-PCR) was performed to evaluate intrarenal AGT and IL-6 mRNA levels using the TaqMan PCR system. Total RNA was isolated using a commercially available RNA isolation kit (Qiagen, Germantown, MD, USA). RNA concentration was quantified using Nanodrop 2000 (Thermo Scientific, Waltham, MA, USA). Subsequently, qRT-PCR was performed, as previously described [7]. All samples were analyzed in triplicate, and the data were normalized based on expression levels of rat β-actin mRNA.

### 4.7. Droplet Digital PCR

We evaluated changes in inflammasome-associated genes, NLRP3 and AIM2, as markers of renal inflammation [47]. NLRP3 inflammasome is activated by ATP-P2Y7 axis and reactive oxygen species, and the expression of pro-NLRP3 is promoted by NF-κB activated by cytokines [47]. Therefore, augmentation of NLRP3 was expected in Ang II-dependent hypertension. On the other hand, AIM2 forms another type of inflammasome complex by viral or bacterial infections [47]. Since their mRNA levels are much lower than AGT and IL-6 mRNA in kidneys, droplet digital PCR (ddPCR) technique, a sensitive gene analysis, was employed. ddPCR was performed using a Bio-Rad ddPCR system as previously described [44]. Primers, probes, and reagents for the One-step RT-ddPCR system were purchased from Bio-Rad to generate cDNA and quantify gene expression. After droplet generation and PCR amplification, droplets were analyzed on the QX200 droplet reader and target cDNA concentration was determined using the QuantaSoft analysis software (Bio-Rad, Hercule, CA, USA). Data are expressed as copy numbers of the target gene in 1 ng total RNA. Experimental and biologic replicates were applied.

### 4.8. Western Blot Analysis

Expression levels of intrarenal AGT were detected using Western blot analysis. In addition, phosphorylation levels of p47 phox, a subunit of NADPH oxidase, in the renal cortex were detected to evaluate renal oxidative stress levels. The Western blot analysis was previously described [7]. The renal cortex was dissected and homogenized with 80 μL of lysis buffer containing 1% Triton X-100, 150 mM NaCl, 1 mM EDTA, 1% Nonidet P-40, 1 mM Na_3_VO_4_, and 0.25% Protease Inhibitor Cocktail (Sigma). The lysates were sonicated 3 times for 10 sec each and centrifuged at 13,000 rpm at 4 °C for 30 min. Total protein concentration of the supernatant was quantified using Micro BCA Protein Assay Kit (Pierce, Singapore, Singapore). Then, 20 µg of total protein was applied to a pre-cast NuPAGE 4–12% gel (Invitrogen, Waltham, MA, USA). The separated proteins were transferred to a nitrocellulose membrane (Bio-Rad). An anti-AGT antibody (1 µg/mL, IBL) and an IRDye labeled anti-goat IgG antibody (1:15,000, Li-Cor, Tokyo, Japan) were used for the detection of AGT. An anti-phospho- p47 phox antibody (Ser370, 1:1000, Assay Biotech, Fremont, CA, USA) and an IRDye labeled anti-goat IgG antibody (1:15,000, Li-Cor) were used to determine phosphorylation levels of p47 phox. Total p47 phox levels were determined using p47 antibody (1:500, Santa Cruz, Dallas, TX, USA). A mouse anti-β-actin antibody (1:1000, Abcam, Cambridge, UK) and an IRDye labeled anti-mouse IgG antibody (1:15,000, Li-Cor) were used for the detection of rat β-actin. Detection was performed using the Odyssey System (Li-Cor). AGT levels were normalized based on β-actin protein levels, and phospho-p47 phox levels were normalized based on a total of p47 phox protein levels.

### 4.9. Histological Analysis

Glomerular mesangial expansion was evaluated by periodic acid-Schiff staining (PAS). Levels of renal tubule interstitial fibrosis were determined by Masson’s trichrome (MT) stain. Kidney tissues were fixed in 10% buffered formalin for 24 h, embedded in paraffin and cut into 4 µm sections. Images were taken in a blinded manner with an Olympus BX51 microscopic system. Twenty glomeruli in a kidney section were randomly selected for PAS scoring. MT scores were obtained from 20 pictures in the renal cortex, as well as in 20 glomeruli for each animal. Image Pro-plus software (Media Cybernetics, Inc., Rockville, MD, USA) was used to mechanically score MT and PAS staining to avoid bias. Randomly selected images for each stain were used to determine threshold levels. Thereafter, fibrosis and mesangial expansion were automatically quantified by the software, based on the previously mentioned thresholding. Fibrosis is expressed as a percentage of the positive area within the total area of the image for the interstitium. Glomerular fibrosis and mesangial expansion are shown as percentages of the positive area within the total area of the glomerulus.

### 4.10. Immunohistological Staining

The AGT and IL-6 protein levels were evaluated by immunostaining. Moreover, levels of CD68 in the renal cortex were analyzed by immunostaining to evaluate immune cell activation and/or infiltration into the kidneys. Formalin fixed kidney sections (4 µm) were deparaffinized with xylene and dehydrated with ethanol. The samples were heated at 100 °C for 60 min in citrate buffer, and target proteins were visualized by using primary antibodies, a Vectastain ABC kit (VECTOR laboratories, Burlingame, CA, USA) and 3,3′-diaminobenzidine substrate kits. Samples were co-stained with hematoxylin before analysis. The staining was performed with an Autostainer Plus (Dako, Carpinteria, Carpinteria, CA, USA). Immunoreactivity was semi-quantitatively evaluated in a blinded test as described in histological analysis section. Density was determined as intensity beyond the threshold of the background signal (darker brown in contrast with lighter brown) and was measured automatically by the Image Pro-plus software (Media Cybernetics, Inc., Rockville, MD, USA). This was then divided by the total area within the image.

### 4.11. Statistical Analysis

Data are expressed as means ± SE. The data were analyzed using Student *t*-test or one-way ANOVA followed by post hoc Tukey multiple comparison test. A value of *p* < 0.05 was considered statistically significant.

## Data Availability

The data presented in this study are available on request from the corresponding author.

## Supplementary Materials

The following supporting information can be downloaded at: https://www.mdpi.com/article/10.3390/ijms23147680/s1.
