# Supplementary material for: Immunosuppression by Mycophenolate Mofetil Mitigates Intrarenal Angiotensinogen Augmentation in Angiotensin II-Dependent Hypertension
Source: Int J Mol Sci. 2022 Jul 12;23(14):7680. doi: 10.3390/ijms23147680 (PMC9319385; doi:10.3390/ijms23147680)
Supplement: Supplementary file 1 [file ijms-23-07680-s001.zip › ijms-1786337-supplementary.pdf]

Supplementary Material

Title

Immunosuppression by Mycophenolate Mofetil Attenuates In-trarenal Angiotensinogen Augmentation in Angiotensin II-Dependent Hypertension

Authors

Ryousuke Satou1, Martha G. Franco2, Dugas M. Courtney1, Akemi Katsurada1, L. Gabriel Navar1

1Department of Physiology, and Hypertension and Renal Center of Excellence, Tulane University School of Medicine, New Orleans, LA

2Departments of Nephrology and Pathology, Instituto Nacional de Cardiologia, Mexico City

Corresponding Author

Ryousuke Satou, PhD

Department of Physiology, and Hypertension and Renal Center of Excellence, Tulane University Health Sciences Center, New Orleans, LA

1430 Tulane Avenue, #SL39/M720, New Orleans, LA 70112-2699, USA

Tel: 504-988-4364, E-mail: rsato@tulane.edu

Expanded Results

Figure S1

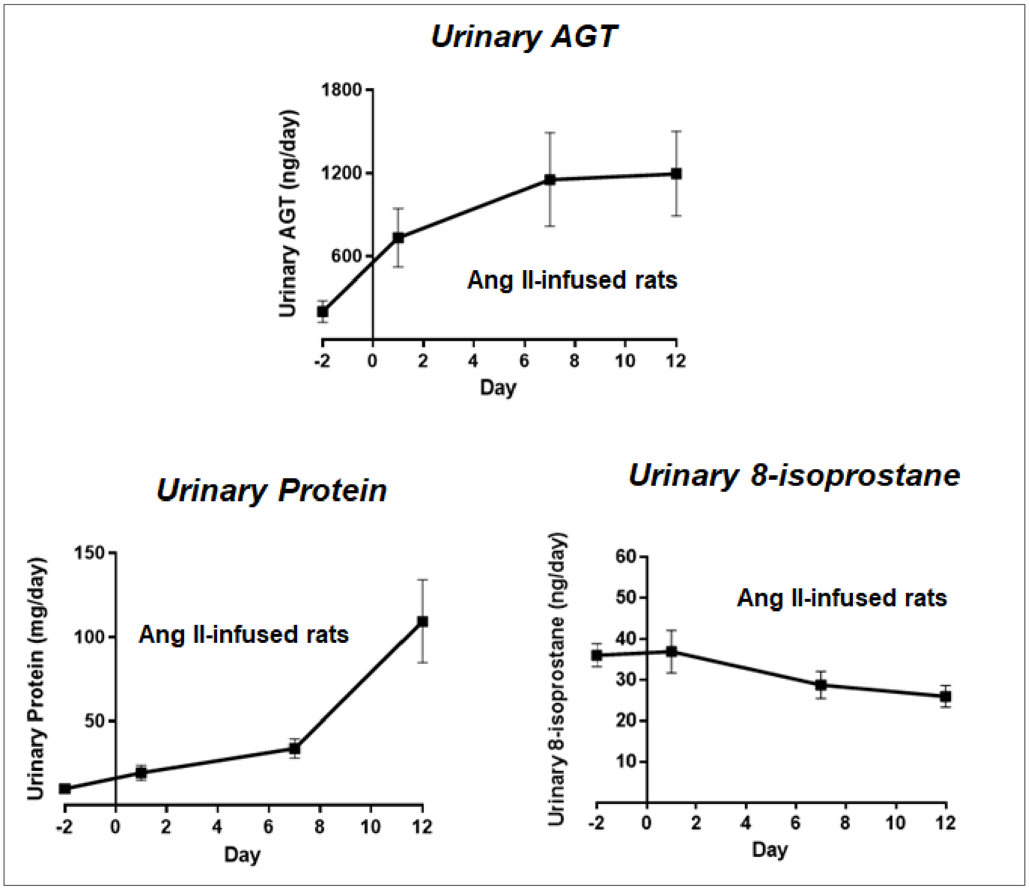

Figure Legend

Figure S1.

Fig. S1. Comparison of temporal changes in urinary AGT, protein and 8-isoprostane levels during Ang II infusion

The figures show temporal changes in urinary AGT, protein and 8-isoprostane levels during Ang II infusion, which are also shown in in Fig. 3D, 4A and 4B.
